# Long-Term Exposures to Air Pollutants and Risk of Peripheral Arterial Occlusive Disease: A Nationwide Cohort Study in Taiwan

**DOI:** 10.3389/fcvm.2022.796423

**Published:** 2022-05-20

**Authors:** Shu-Hui Liao, Chien-Shan Chiu, Li-Ho Jang, Sung-Yuan Hu, Chorng-Kuang How, Vivian Chia-Rong Hsieh, Ming-Shun Hsieh

**Affiliations:** ^1^Department of Pathology and Laboratory, Taipei Veterans General Hospital, Taoyuan Branch, Taoyuan, Taiwan; ^2^Department of Dermatology, Taichung Veterans General Hospital, Taichung, Taiwan; ^3^Institute of Biomedical Sciences, National Chung Hsing University, Taichung, Taiwan; ^4^Department of Critical Care, Saint Paul's Hospital, Taoyuan, Taiwan; ^5^Department of Emergency Medicine, Taichung Veterans General Hospital, Taichung, Taiwan; ^6^School of Medicine, Chung Shan Medical University, Taichung, Taiwan; ^7^Institute of Medicine, Chung Shan Medical University, Taichung, Taiwan; ^8^Department of Post-Baccalaureate Medicine, College of Medicine, National Chung Hsing University, Taichung, Taiwan; ^9^School of Medicine, National Yang Ming Chiao Tung University, Taipei, Taiwan; ^10^Department of Emergency Medicine, Taipei Veterans General Hospital, Taipei, Taiwan; ^11^Department of Health Services Administration, China Medical University, Taichung, Taiwan; ^12^Department of Emergency Medicine, Taipei Veterans General Hospital, Taoyuan Branch, Taoyuan, Taiwan

**Keywords:** peripheral arterial occlusive disease (PAOD), PM_2.5_, carbon monoxide (CO), nitrogen dioxide (NO_2_), air pollutants, prolonged exposure

## Abstract

Air pollution is one of the most alarming environmental issues which causes multiple health hazards. An association between air pollution and cardiovascular diseases has been established through many prior studies. In this study, we aimed to evaluate the risk of long-term exposure to air pollution (PM_2.5_, CO, and NO_2_) and its association with the risk of developing peripheral arterial occlusive disease (PAOD). PAOD is a condition involving impairment of perfusion of blood in the distal parts of the aorta due to narrowing of the arteries (arterial stenosis) and has been reported as a risk factor for developing cardiovascular diseases. Furthermore, the risk of PAOD increases with age, and hence is a serious public health issue and a cause for concern, especially for an aging society such as Taiwan. Two national-scale databases from Taiwan, the national health insurance database (NHIRD) and the Taiwan air quality-monitoring database (TAQMD), were linked to conduct this cohort study between 2003 and 2013. Cox proportional hazards regression with time-dependent modeling was used to evaluate the hazard ratio (HR) for PAOD with respect to daily exposure to air pollutants. The concentrations of each of the pollutants of interest (PM_2.5_, NO_2_, and CO) were categorized into four categories according to the daily average concentration of air pollutants for every quarter of the year, Q1 to Q4 (Q4 = highest). The cumulative incidence of PAOD was examined by Kaplan–Meier analysis with two-tailed log-rank test. A total of 1,598 PAOD cases were identified during the 10-year follow-up period, along with 98,540 non-PAOD controls. In the multivariate analysis, after adjusting for age, gender, urbanization level, residential area, baseline comorbidities, and medications, the adjusted HRs were PM_2.5_ = 1.14 (95% CI 1.13–1.16), NO_2_ = 1.03 (95% CI 1.02–1.04), and CO = 2.35 (95% CI 1.95–2.84). Kaplan–Meier analysis showed that CO (*P* < 0.0001) and PM_2.5_ (*P* < 0.0001) concentrations were strongly and positively associated with the cumulative incidence of PAOD during the follow-up period. Findings from this study established that prolonged exposure to air pollutants CO and PM_2.5_ are significant factors that, among other well-known causes, may also play a potential role in PAOD pathogenesis.

## Introduction

Air pollution is one of the most alarming environmental issues which causes of multiple health hazards and ranks ninth among the modifiable disease risk factors, preceding other frequent factors such as low physical activity, a high-sodium diet, high cholesterol, and drug use. Air pollution is responsible for 3.1% of global disability-adjusted life years (DALY), an index that quantifies the time spent under reduced health conditions (1). Numerous studies on Caucasians, Black Americans, Europeans, and Asians have established that air pollutants, including gaseous compounds such as carbon monoxide (CO), nitrogen dioxide (NO_2_), sulfur dioxide (SO_2_), and ozone (O_3_), and particulate matter (PM) such as PM_10_ and PM_2.5_, are associated with poorer cardiovascular (CV) conditions, and that exposure to air pollutants can increase the risk of CV events (1–3). The major cause for such CV morbidity is mainly due to coronary artery diseases that develop as an effect of continued exposure to air pollution. A multicity study from various countries (Europe, the United States, and Canada) has reported an increase of up to 0.6% in all-cause mortality with every 10 μg/m^3^ increase in PM_10_ (4). Another project, the Air Pollution and Health: a European Approach (APHEA) study (5), conducted in 30 European cities, reported that a 10 μg/m^3^ increase in NO_2_ led to an up to 0.4% increase in total, CV, and respiratory mortality rates. Further, studies on European cohorts also demonstrated the effects of PM_2.5_ on CV mortality for both outdoor and indoor subjects. Studies on the long-term effects of air pollution in the Asian countries are scarcely found. A study on the Taiwanese population reported a positive correlation of the risk of all-cause and cardiovascular disease (CVD) mortality with levels of SO_2_, CO, NO_2_, and PM_10_ especially for the elderly (age > 65 years) (6). Air pollution in Taiwan, due to gaseous pollutants and PM_2.5_ and PM_10_, is categorized as either local pollution, the source being motor vehicles and power plants, or long-range transported pollution, the source being neighboring countries, and is an acute issue that has been a cause for great concern among laypersons and researchers alike (7).

Peripheral arterial occlusive disease (PAOD) is a condition involving impaired perfusion of blood in the distal parts of the aorta and/or the pelvic, femoral, and crural arteries due to narrowing of the arteries (arterial stenosis) or absolute arterial lumen blockage (occlusion). It has been reported as a risk factor for developing CVD (8, 9). PAOD has a rising incidence and currently affects ~200 million people worldwide (estimated prevalence rose from 164 million in 2000 to 201 million in 2010) (8). In the Asia-Pacific region, PAOD prevalence ranges between 5.2 and 12.1% (10). A systematic review of 34 countries revealed that its prevalence in men in high-income countries was higher than that in low and middle-income countries; however, incidence in women was found to be uniform (11). Moreover, it was reported that the overall prevalence of PAOD has increased by 13.1% in high-income countries and by 28.7% in low- and middle-income countries. PAOD diminishes quality of life. Approximately 50% of PAOD patients are asymptomatic, therefore preventing an accurate estimation of its true prevalence (12). Furthermore, PAOD mostly occurs in people with atherosclerosis; therefore, it is an important indicator of atherosclerotic burden (13), and has been reported in multiple studies to have an association with an enhanced risk of mortality due to CV conditions (14, 15). As the risk of PAOD increases with age (9), it is a serious public health issue, and a cause for concern, especially for an aging society such as Taiwan (16). With the exception of a few studies on Asian cohorts, PAOD has not received enough attention compared with CVD, particularly in the context of risk of developing it as a result of prolonged exposure to pollution (17, 18). Therefore, this study focuses on evaluating the contribution of long-term exposure to air pollution (PM_2.5_, CO, and NO_2_) to the development of PAOD, using individual-level data from national databases in Taiwan.

## Materials and Methods

### Databases

Two national-scale databases from Taiwan, the National Health Insurance Research Database (NHIRD) and the Taiwan Air Quality-Monitoring Database (TAQMD), were used to obtain individual clinical data and environmental data, respectively, between 2003 and 2013. NHIRD is a large-scale administrative healthcare database consisting of medical records, causes of death records, sorted registration files, and original claims data that are derived from the National Health Insurance (NHI) program by the NHI Bureau and is maintained by the National Health Research Institutes (NHRI). It houses information on 23 million residents of Taiwan covering 99% of the total population that is available to scientists in Taiwan for research purposes (19). TAQMD is a large-scale database that is set up by Taiwan's official Environmental Protection Administration (EPA) and Central Weather Bureau (CWB) to document and forecast air quality. The database consists of ~260,000 samples that were retrieved in 2017 from 77 air monitoring stations (EPA) and 580 weather stations (CWB) in Taiwan, and the data are freely available for public access (20, 21). Both databases were utilized to obtain linked information for each of the individuals in this study.

### Study Population

**Figure 1:**
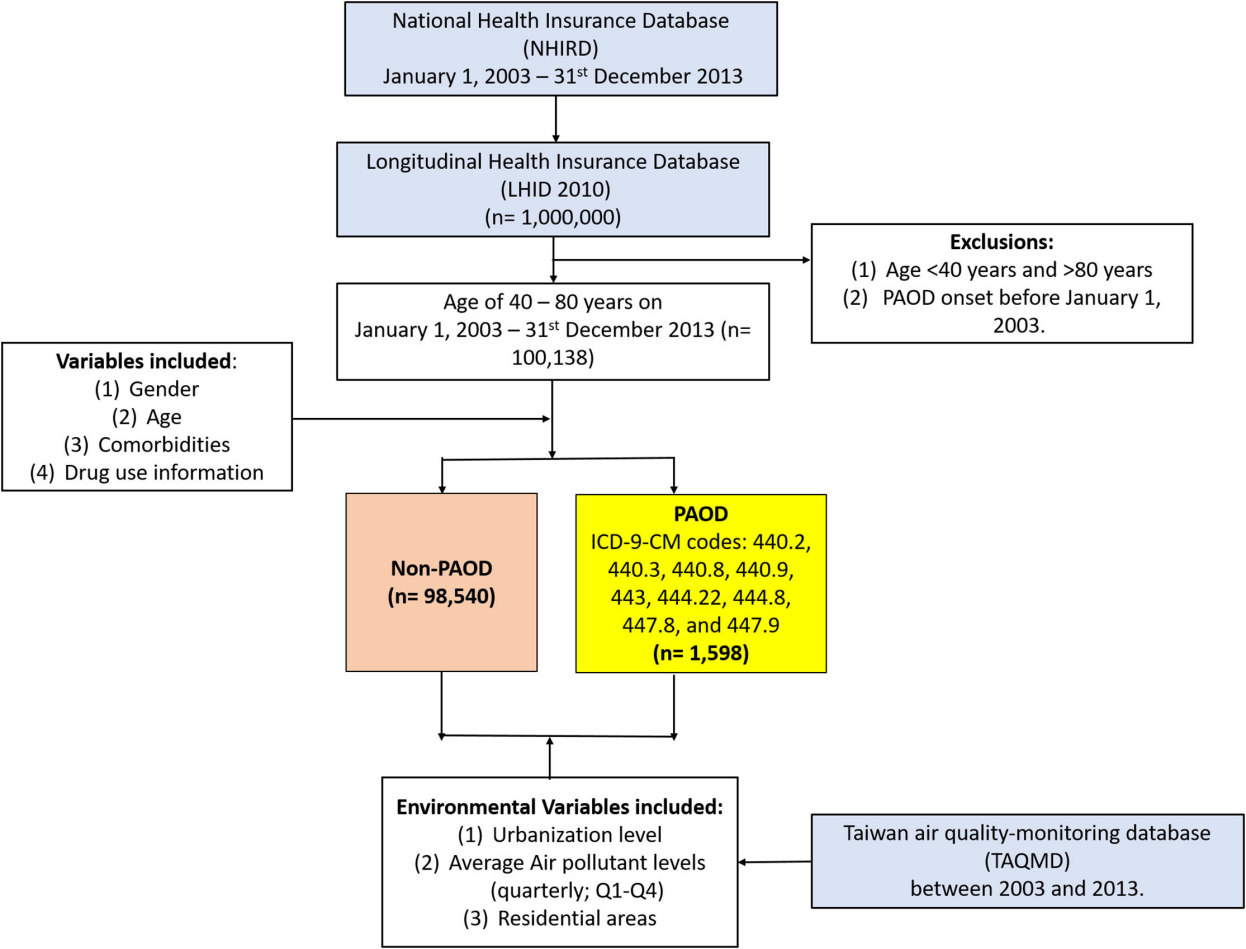
The selection of the subjects included in the study.

A total of 1,000,000 individuals from the longitudinal component of the NHIRD (19) were selected, among whom only those aged 40–80 years as on 1 January 2003, were included in this study. A total of 1,598 individuals were diagnosed with PAOD as per International Classification of Diseases, Ninth Revision, Clinical Modification (ICD-9-CM) codes 440-447, with first onset on or after 1 January 2003, and 98,540 without PAOD were retained for further analysis (Figure 1). Patients with a history of PAOD prior to the index date (1 January 2003) or those who were <40 years of age were excluded from further analysis. All patients were followed until 31 December 2013. The primary outcome was defined as the first PAOD event within the follow-up period of 10 years.

### Clinical Variables

Gender, age, comorbidities, and medication usage information were included as clinical variables in this study (Figure 1). Four age groups (40–49, 50–59, 60–69, 70–80 years) were considered. Comorbidities were considered if individuals were diagnosed with any other related condition(s) within 1 year prior to the beginning of the study period. Comorbidities due to hypertension (ICD-9-CM: 401–405), diabetes mellitus (DM) (ICD-9-CM: 250.x), hyperlipidemia (ICD-9-CM: 272, A182), chronic obstructive pulmonary disease (COPD) (ICD-9-CM:490, 491, 492, 494, 496), congestive heart failure (CHF) (ICD-9-CM: 428), chronic kidney disease (CKD) (ICD-9-CM: 581–588, 403–404, 285.21), cerebral vascular disease (stroke) (ICD-9-CM: 430–438), ischemic heart disease (IHD) (ICD-9-CM: 411–414), and cancer (ICD-9-CM: 140–208) were included as variables in this study. Information on usage of medications, for each individual, was taken into consideration if particular medicines were used for more than a month during the study period. Medications such as statins, aspirin, clopidogrel, angiotensin-converting enzyme inhibitors (ACEIs), angiotensin receptor blockers (ARBs), warfarin (anticoagulants), and steroids were included in the analysis.

### Environmental Variables

Environmental variables such as urbanization level, residential areas, and level of air pollution were included. The NHRI has stratified all city districts and townships in Taiwan into seven urbanization levels (levels 1–7) on the basis of (1) population density (people/km^2^), (2) the proportion of residents with higher education level, (3) occupation types (e.g., who work in agriculture), and (4) the number of physicians per 100,000 people in each area. Level 1 represents areas with a low population density and socioeconomic status and level 7 represents the highest socioeconomic status. As very few people reside in the rural areas that predominantly fall under classification 1–3, these areas were regrouped as level 1. Therefore, the urbanization level was reclassified into 5 levels. Air pollution levels were determined based on the level of exposure to pollutant, which was defined with respect to time period (T) and the concentration of pollutants to which an individual was exposed. The pollution data were collected every day of the year and the daily average was obtained over each year of the study period, which were linked with each of the individuals in this study. The concentrations of each of the pollutants of interest (PM_2.5_, NO_2_ and CO) were categorized into four groups based on the following thresholds: PM_2.5_ (Q1 = <28.24 μg/m^3^/day, Q2 = 28.24–31.46 μg/m^3^/day, Q3 = 31.46–38.47 μg/m^3^/day, and Q4 ≥ 38.47 μg/m^3^/day); NO_2_ (Q1 <16.14 ppb/day, Q2 = 16.14–20.49 ppb, Q3 = 20.49–24.90 ppb/day, and Q4 ≥ 24.90 ppb/day); and CO (Q1 <0.47 ppm/day, Q2 = 0.47–0.58 ppm, Q3 = 0.58–0.68 ppm/day, and Q4 ≥ 0.68 ppm/day). Residential areas for patients were broadly divided into six distinct regions, namely, (i) Northern, (ii) Taipei, (iii) Central, (iv) Southern, (v) Eastern, and (vi) Kao-Ping, based on the location where the most clinic and hospital visits were made for treating acute upper respiratory tract infections, as defined by ICD-9-CM code 460.

### Statistical Analysis

All baseline variables, both clinical and environmental, were comparatively analyzed between individuals with PAOD and without PAOD, using chi-square tests or the means of independent samples *t*-test (22). First, univariate Cox proportional hazard regression analysis (23) was conducted with the PAOD event as the outcome. The crude hazard ratios (HRs) (univariate) for each of the air pollutants (PM_2.5_, NO_2_, and CO), gender, age group, urbanization level, residential area, comorbidities, and drug use were calculated. Then, three different Cox proportional hazards regression analyses with time-dependent covariates (24) were performed with the first PAOD event as the primary outcome for each of the pollutants (PM_2.5_, NO_2_, and CO), adjusted by the segmented time-dependent covariates, gender, age group, urbanization level, residential area, comorbidities, and drug use. This was done to avoid bias due to non-baseline variables that changed over the course of time in combination with time to event. Two-tailed log-rank tests were used to obtain *p*-values, and a threshold of *p* < 0.05 was used to define statistical significance. Further, the residential areas were divided into four categories according to the daily average concentration of air pollutants, Q1–Q4 (Q4 = highest) and were used to examine the cumulative incidence of PAOD, based on each of the pollutants under study (PM_2.5_, CO, NO_2_), through Kaplan–Meier analysis with a two-tailed log-rank test (25). Again, a *p*-value threshold of 0.05 was used to define statistical significance. All analyses were conducted using SAS 9.4 (SAS Institute Inc., Cary, NC, USA).

## Results

### Clinical Characteristics and Linked Environmental Exposure Details

After exclusion due to prior PAOD diagnosis and age <40 years and >80 years, a total of 100,138 individuals were included in the final analysis, among whom 1,598 (1.6%) and 98,540 (98.4%) were with and without PAOD, respectively (Figure 1). Table 1 gives a detailed account of all demographics, clinical variables, and environmental variables included for analysis in this study. Significant differences for all baseline variables were observed between individuals with and without PAOD. Men made up the majority of PAOD cases (53.32%), whereas women comprised the majority (54.79%) of those without PAOD. The mean age of individuals with PAOD was 63.22 years, the incidence being higher among older individuals. The highest number of events was observed in the age group 70–80 years (32.29%), followed by 60–69 years (32.1%), 50–59 years (21.84%), and 40–49 years (13.77%), while the trend was reverse for people with no PAOD. A majority of people with (64.14%) and without PAOD (66.84%) were from the top two urbanized zones (level 4 and level 5), and 69.33% of people with PAOD were from the northern areas of Taiwan, the highest number being from Taipei (37.17%). Comorbidities due to all related conditions were significantly different among people with and without event, with hypertension (42.74%) and DM (30.6%) being present in highest number of PAOD patients, and cancer (3.13%) and CHF (3%) in the lowest number of patients. A similar trend was observed for people without PAOD; however, the proportions of individuals with related conditions were significantly lower. Finally, drug usage was also found to be significantly different between individuals with and without PAOD. Most patients with PAOD were observed to be on drugs for reducing the risk of CVD, such as ARBs, aspirin, ACEIs, and statins. Therefore, to avoid bias due to time-dependent non-baseline variables (exposure to pollution) that changed along with time to event, the fitted models were adjusted by all baseline variables as covariates.

**Table 1:** Demographic characteristics of PAOD cases and non-PAOD subjects.

**Variables**	**PAOD**				*p*-value*
	**No (*****n*** **=** **98,540, 98.40%)**		**Yes (*****n*** **=** **1,598, 1.60%)**		
	***N***	**%**	***n***	**%**	
**Gender**					<0.0001
Female	53,986	54.79	746	46.68	
Male	44,554	45.21	852	53.32	
**Age group**					<0.0001
40–49	45,593	46.27	220	13.77	
50–59	27,017	27.42	349	21.84	
60–69	16,129	16.37	513	32.1	
70–80	9,801	9.95	516	32.29	
Mean ± SD (years)^†^	53.55 (10.31)		63.22 (10.34)		<0.0001
**Urbanization level**					<0.0001
1 (lowest)	6,769	6.87	152	9.51	
2	10,711	10.88	205	12.83	
3	15,179	15.41	216	13.52	
4	32,477	32.97	533	33.35	
5 (highest)	33,355	33.87	492	30.79	
**Residential area**					0.0022
Northern	14,996	15.22	269	16.83	
Taipei	37,960	38.52	594	37.17	
Central	14,960	15.18	245	15.33	
Southern	8,795	8.93	168	10.51	
Eastern	2,962	3.01	62	3.88	
Kao-Ping	18,867	19.15	260	16.27	
**Comorbidities**					
Hypertension	17,387	17.64	683	42.74	<0.0001
DM	7,506	7.62	489	30.6	<0.0001
Hyperlipidemia	6,547	6.64	221	13.83	<0.0001
COPD	5,152	5.23	178	11.14	<0.0001
CHF	850	0.86	48	3	<0.0001
CKD	1,920	1.95	117	7.32	<0.0001
Stroke	2,706	2.75	172	10.76	<0.0001
IHD	5,280	5.36	249	15.58	<0.0001
Cancer	2,208	2.24	50	3.13	0.0177
**Drug use**					
Statin	29,078	29.51	589	36.86	<0.0001
Aspirin	21,779	22.1	651	40.74	<0.0001
Clopidogrel	4,771	4.84	220	13.77	<0.0001
ACEI	22,942	23.28	640	40.05	<0.0001
ARB	30,617	31.07	718	44.93	<0.0001
Warfarin	1,826	1.85	74	4.63	<0.0001
Steroid	27,110	27.51	392	24.53	0.0081

*ACEI, Angiotensin-converting enzyme inhibitors; ARB, Angiotensin receptor blockers; CHF, Congestive heart failure; CKD, Chronic kidney disease; COPD, Chronic obstructive pulmonary disease; DM, Diabetes mellitus; IHD, Ischemic heart disease; PAOD, Peripheral artery occlusive disease; SD, Standard deviation.*

*^†^Two-sample t-test*.

### Survival Analysis

**Figure 2:**
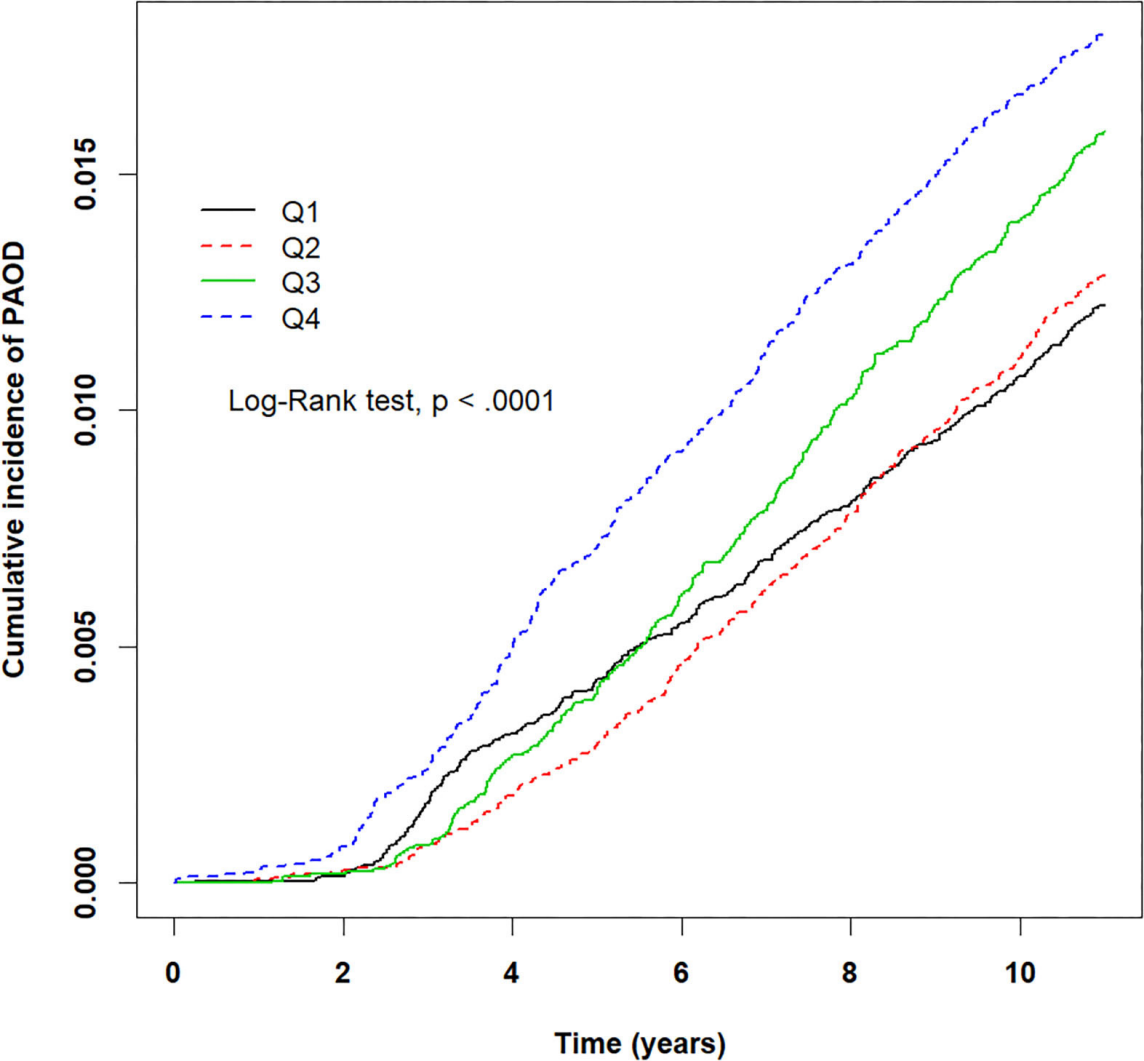
Kaplan–Meier plots for different levels (Q1–Q4) of prolonged exposure to particulate matter PM_2.5_ and its association with cumulative incidence rate of peripheral arterial occlusive disease (PAOD) during a 10-year follow-up period. X-axis displays the time in years. Y-axis displays the cumulative incidence of PAOD. Q1: PM_2.5_ < 28.24 μg/m^3^, Q2: 28.24 ≤ PM_2.5_ < 31.46 μg/m^3^, Q3: 31.46 ≤ PM_2.5_ < 38.47 μg/m^3^, and Q4: PM_2.5_ ≥ 38.47 μg/m^3^. *p* < 0.05 is the threshold for significance.

**Figure 3:**
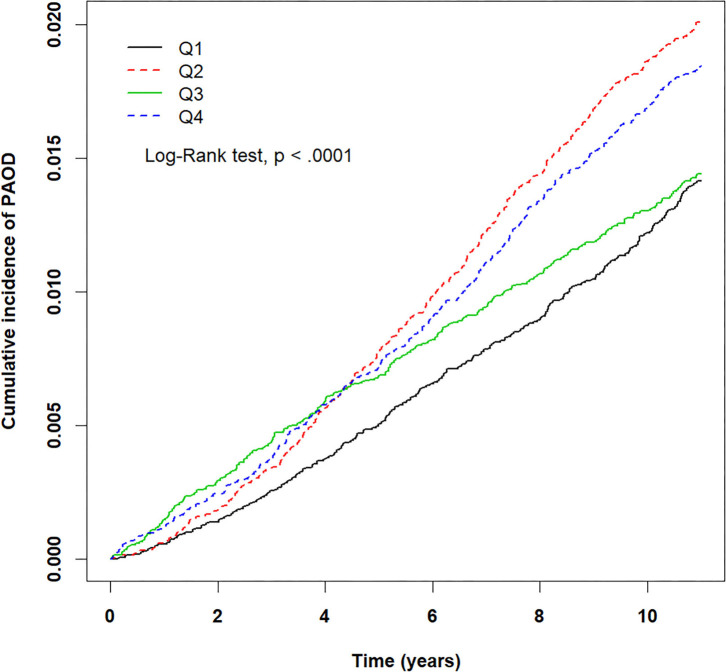
Kaplan–Meier plots for different levels (Q1–Q4) of prolonged exposure to nitrogen dioxide (NO_2_) and its association with cumulative incidence rate of peripheral arterial occlusive disease (PAOD) during a 10-year follow-up period. X-axis displays the time in years. Y-axis displays the cumulative incidence of PAOD. Q1: NO_2_ < 16.14 ppb, Q2: 16.14 ≤ NO_2_ < 20.49 ppb, Q3: 20.49 ≤ NO_2_ < 24.90 ppb, and Q4: NO_2_ ≥ 24.90 ppb. *p* < 0.05 is the threshold for significance.

**Figure 4:**
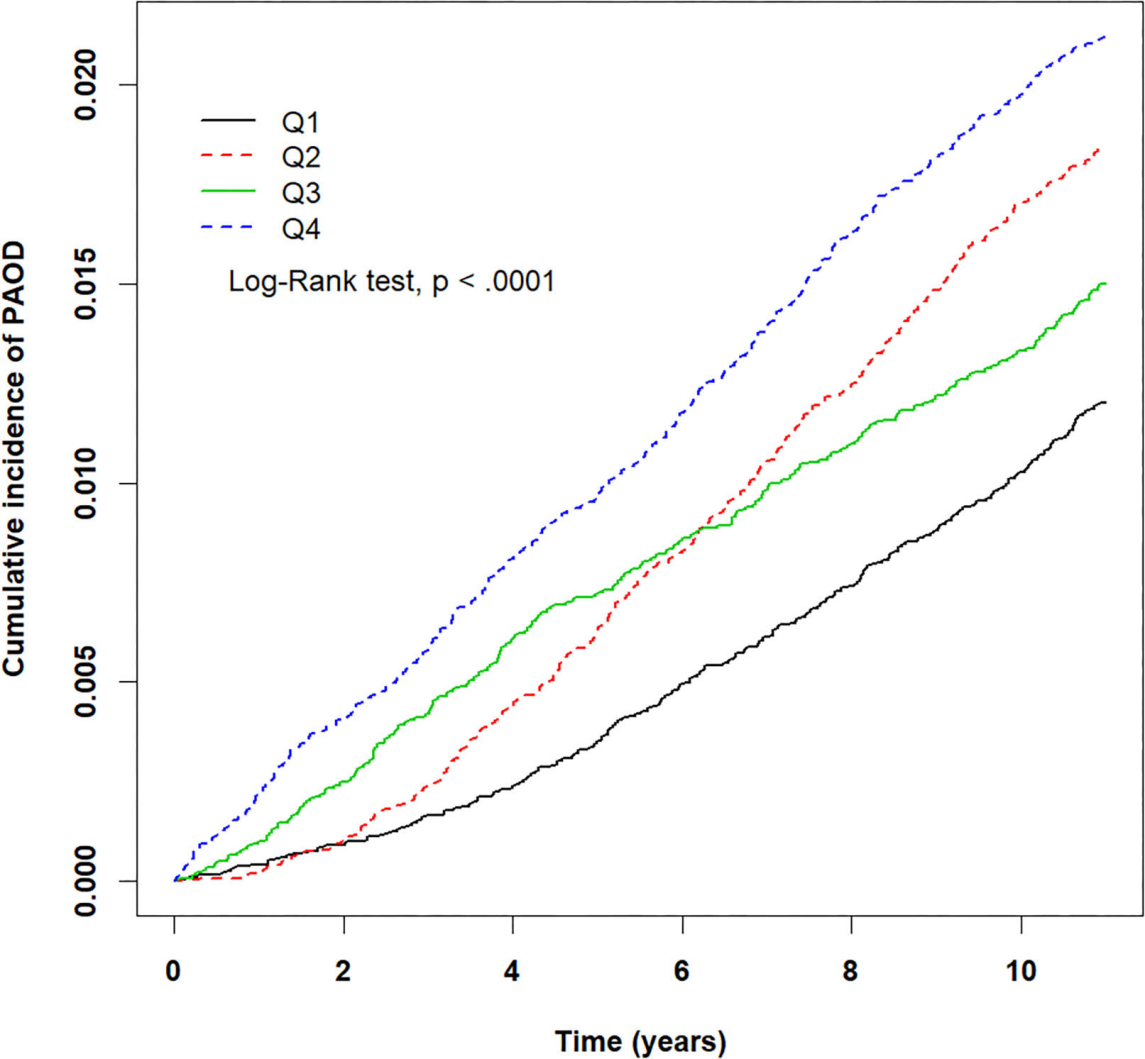
Kaplan–Meier plots for different levels (Q1–Q4) of prolonged exposure to carbon monoxide (CO) and its association with cumulative incidence rate of peripheral arterial occlusive disease (PAOD) during a 10-year follow-up period. X-axis displays the time in years. Y-axis displays the cumulative incidence of PAOD. Q1: CO < 0.47 ppm, Q2: 0.47 ≤ CO < 0.58 ppm, Q3: 0.58 ≤ CO < 0.68 ppm, and Q4: CO ≥ 0.68 ppm. *p* < 0.05 is the threshold for significance.

The crude HRs for both pollutants (PM_2.5_, CO, NO_2_) and baseline variables were obtained using univariate Cox proportional hazards regression models with PAOD event as the outcome. Table 2 displays the results of the crude HRs and corresponding 95% confidence intervals (95% CIs); all variables were found to be statistically significant through log-rank tests, except certain urbanization levels and certain residential areas (Taipei was significant). Notably, individuals from higher urbanized regions (levels 1 and 2) were observed to be at a significantly higher risk of PAOD incidence in comparison to those from the least urbanized region (level 5) (Table 2). Next, each of the pollutants were entered into a multivariate model as a continuous, segmented time-dependent variable, adjusted by all baseline variables as covariates. The results displayed that each of the pollutants, PM_2.5_ (HR = 1.14; 95% CI = 1.13–1.16; *p*-value < 0.0001), NO_2_ (HR = 1.03, 95% CI = 1.02–1.04, *p*-value < 0.0001), and CO (HR = 2.35, 95% CI = 1.95–2.84, *p*-value < 0.0001) had a significant positive effect on the risk of PAOD (Table 2). Furthermore, for the purpose of illustration, Figures 2–4 show the Kaplan–Meier plots wherein the broadly categorized residential areas were used for predicting PAOD incidence over a 10-year follow-up period. The Kaplan–Meier analysis showed that the most polluted area (Q4) had the highest cumulative incidence of PAOD with prolonged exposure to CO followed by areas Q2, Q3, and Q1 (Figure 4). Similarly, prolonged exposure to PM_2.5_ led to the highest cumulative incidence of PAOD for area Q4 followed by Q3, Q2, and Q1 (Figure 2). However, for NO_2_, the highest cumulative incidence of PAOD was not observed among residents from the worst polluted area but for Q2 residential area followed by Q4, Q3, and Q1 (Figure 3). The log-rank tests further confirmed that pollution level (Q1–Q4) was a significant predictor (CO: *p*-value < 0.0001, PM_2.5_: *p*-value < 0.0001, and NO_2_: *p*-value < 0.0001) of cumulative incidence of PAOD, and the findings concluded that CO and PM_2.5_ concentrations were strongly positively associated with the cumulative incidence rate of PAOD during the 10-year follow-up period.

**Table 2:** Cox model with hazard ratio and 95% confidence interval of PAOD in patients exposed to various daily average concentration of air pollutants.

**Variables**	**Crude**		**Adjusted model 1**		**Adjusted model 2**		**Adjusted model 3**	
	**HR (95% CI)**	***p*-value**	**HR (95% CI)**	***p*-value**	**HR (95% CI)**	***p*-value**	**HR (95% CI)**	***p*-value**
PM_2.5(_μg/m^3^), daily average	1.02 (1.02–1.03)	<0.0001	1.14 (1.13–1.16)	<0.0001	–	–	–	–
NO_2_ (ppb), daily average	1.01 (1.00–1.02)	0.0029	–	–	1.03 (1.02–1.04)	<0.0001	–	–
CO (ppm), daily average	1.71 (1.44–2.03)	<0.0001	–	–	–	–	2.35 (1.95–2.84)	<0.0001
**Gender**								
Female	1.00		1.00		1.00		1.00	
Male	1.42 (1.29–1.57)	<0.0001	1.32 (1.19–1.47)	<0.0001	1.29 (1.17–1.42)	<0.0001	1.29 (1.17–1.43)	<0.0001
**Age group**								
40–49	1.00		1.00		1.00		1.00	
50–59	2.7 (2.28–3.2)	<0.0001	2.28 (1.9–2.72)	<0.0001	2.4 (2.03–2.85)	<0.0001	2.4 (2.02–2.84)	<0.0001
60–69	6.88 (5.88–8.06)	<0.0001	4.69 (3.93–5.59)	<0.0001	5.07 (4.29–6)	<0.0001	5.03 (4.25–5.95)	<0.0001
70–80	12.88 (11–15.09)	<0.0001	7.18 (5.96–8.64)	<0.0001	7.87 (6.61–9.38)	<0.0001	7.85 (6.59–9.36)	<0.0001
**Urbanization level**								
1 (lowest)	1.00		1.00		1.00		1.00	
2	1.12 (0.99–1.27)	0.0643	1.15 (0.99–1.33)	0.0598	1.16 (1.01–1.32)	0.038	1.15 (1.01–1.32)	0.0381
3	0.98 (0.83–1.15)	0.7714	0.99 (0.83–1.2)	0.953	1.04 (0.87–1.23)	0.6879	1.05 (0.89–1.25)	0.5497
4	1.32 (1.12–1.56)	0.0008	1.45 (1.19–1.76)	0.0002	1.28 (1.07–1.55)	0.0084	1.24 (1.03–1.49)	0.0209
5 (highest)	1.57 (1.31–1.89)	<0.0001	1.23 (0.99–1.52)	0.0564	1.39 (1.13–1.71)	0.0016	1.36 (1.11–1.67)	0.0032
**Residential area**								
Northern	1.00		1.00		1.00		1.00	
Taipei	0.87 (0.75–1)	0.0512	1.17 (0.99–1.39)	0.071	0.91 (0.77–1.06)	0.2197	0.88 (0.75–1.03)	0.1127
Central	0.92 (0.77–1.09)	0.3192	0.38 (0.31–0.47)	<0.0001	1.02 (0.86–1.22)	0.8152	1.06 (0.88–1.26)	0.5497
Southern	1.08 (0.89–1.31)	0.4416	0.23 (0.18–0.3)	<0.0001	1.13 (0.92–1.39)	0.2342	1.15 (0.94–1.41)	0.1729
Eastern	1.19 (0.9–1.57)	0.2213	5.16 (3.71–7.16)	<0.0001	1.4 (1.04–1.89)	0.0288	1.19 (0.89–1.59)	0.2328
Kao-Ping	0.78 (0.66–0.92)	0.0038	0.09 (0.07–0.13)	<0.0001	0.77 (0.65–0.92)	0.0031	0.8 (0.67–0.95)	0.0103
**Comorbidities (ref** **=** **none)**								
Hypertension	3.67 (3.32–4.05)	<0.0001	1.35 (1.19–1.55)	<0.0001	1.47 (1.3–1.67)	<0.0001	1.47 (1.3–1.66)	<0.0001
DM	5.75 (5.17–6.39)	<0.0001	2.85 (2.51–3.25)	<0.0001	3.16 (2.8–3.57)	<0.0001	3.17 (2.81–3.57)	<0.0001
Hyperlipidemia	2.26 (1.96–2.61)	<0.0001	1.04 (0.89–1.23)	0.6106	1.06 (0.91–1.24)	0.4352	1.07 (0.92–1.25)	0.4022
COPD	2.48 (2.13–2.9)	<0.0001	1.22 (1.02–1.46)	0.0332	1.36 (1.16–1.6)	0.0002	1.36 (1.16–1.6)	0.0002
CHF	4.43 (3.32–5.9)	<0.0001	1.44 (1.04–2.01)	0.0286	1.42 (1.06–1.91)	0.0207	1.42 (1.05–1.91)	0.0214
CKD	4.46 (3.69–5.38)	<0.0001	2.05 (1.66–2.54)	<0.0001	2.13 (1.76–2.58)	<0.0001	2.14 (1.76–2.59)	<0.0001
Stroke	4.93 (4.21–5.78)	<0.0001	1.51 (1.25–1.82)	<0.0001	1.6 (1.35–1.89)	<0.0001	1.58 (1.34–1.87)	<0.0001
IHD	3.47 (3.03–3.97)	<0.0001	1.1 (0.93–1.29)	0.2803	1.22 (1.05–1.42)	0.0084	1.21 (1.05–1.41)	0.0109
Cancer	1.69 (1.28–2.24)	0.0003	1.04 (0.76–1.41)	0.8184	1 (0.75–1.33)	0.9989	1 (0.76–1.33)	0.9851
**Drug use (ref** **=** **none)**								
Statin	1.35 (1.22–1.49)	<0.0001	0.87 (0.77–0.98)	0.0214	0.79 (0.71–0.89)	<0.0001	0.79 (0.71–0.89)	<0.0001
Aspirin	2.46 (2.23–2.72)	<0.0001	1.3 (1.16–1.47)	<0.0001	1.22 (1.09–1.36)	0.0005	1.22 (1.09–1.36)	0.0005
Clopidogrel	3.19 (2.76–3.67)	<0.0001	1.38 (1.17–1.62)	<0.0001	1.27 (1.09–1.48)	0.0027	1.26 (1.08–1.48)	0.003
ACEI	2.23 (2.02–2.47)	<0.0001	1.15 (1.01–1.3)	0.0314	1.08 (0.96–1.21)	0.2177	1.08 (0.96–1.21)	0.2235
ARB	1.78 (1.62–1.97)	<0.0001	0.85 (0.75–0.96)	0.009	0.72 (0.64–0.81)	<0.0001	0.72 (0.64–0.81)	<0.0001

*HR, hazard ratio; CI, confidence interval; CEI, Angiotensin-converting enzyme inhibitors; ARB, Angiotensin receptor blockers; CHF, Congestive heart failure; CKD, Chronic kidney disease; COPD, Chronic obstructive pulmonary disease; DM, Diabetes mellitus; IHD, Ischemic heart disease; PAOD, Peripheral artery occlusive disease; SD, Standard deviation; Model 1: adjusted for PM_2.5_, age, gender, urbanization level, residential area, comorbidity, and drugs in Cox proportional hazards regression. Model 2: adjusted for NO_2_, age, gender, urbanization level, residential area, comorbidity, and drugs in Cox proportional hazards regression. Model 3: adjusted for CO, age, gender, urbanization level, residential area, comorbidity, and drugs in Cox proportional hazards regression*.

## Discussion

This study utilizes two national-scale databases from Taiwan and demonstrated that the risk of long-term exposure to PM_2.5_ and CO has a significant positive association with the cumulative risk of incidence of PAOD, through a 10-year follow-up period. Higher risks of PAOD were reported for individuals residing in the most urbanized regions as opposed to those in lower urbanized areas (levels 1 and 2). Moreover, men were at a higher risk than women. Factors such as age, comorbidities, and usage of medications were also demonstrated to impose a higher risk of developing PAOD for individuals with long-term exposure to air pollutants. Hence, this is an important study that highlights the significant adverse effect that environmental factors have on the development of PAOD, a topic that has not received the attention that it warrants.

MESA air (Multi-Ethnic Study of Atherosclerosis and Air Pollution) studies were conducted extensively on participants from six US communities, utilizing high performance pollution exposure models to understand the association between environmental exposures and a wide range of CV-related outcomes. The series of studies provided knowledge about the impact of air pollutants on CV disease thereby offering a research paradigm for improving and developing environmental epidemiology (26–28). However, very few Asian studies exist in the literature that deals with the health effects of air pollution. One such study was conducted on national-level data from Korea; however, it reported that long exposure to SO_2_ and NO_2_ data was an independent risk factor for PAOD, whereas new incident cases were unaffected by exposure to particulate matter, such as PM_2.5_ and PM_10_ (29). Another study reported a positive association of exposure to ozone (O_3_) and CO (air pollution) with the number of emergency admissions for cerebrovascular disease among adults in Taipei (30). Despite its poor prognosis, PAOD is majorly underdiagnosed, underestimated, and undertreated. It has been claimed that there may be ethnic and region-based differences in the Asia-Pacific in terms of epidemiological resources, availability of diagnostic and therapeutic modalities, and patient treatment response. This is why the Asian Pacific Society of Atherosclerosis and Vascular Diseases (APSAVD) initiated an Asia-Pacific Consensus Statement (APCS) for managing and raising awareness for PAOD (10). This study may provide insights into the mechanisms for the development of PAOD, which may then lead to awareness in the general population regarding one of the major risk factors for PAOD incidence among Asians.

CO exposure was found to have a positive and strong association with incident PAOD among the Taiwanese population. CO binds irreversibly to hemoglobin with a binding affinity 200–400 times to that of oxygen, thus leading to hypoxia and tissue damage. Moreover, long-term CO exposure could amount to an enhanced thickening of the carotid intima-media and elevated serum C-reactive protein, thereby conferring a higher risk for patients to develop CVD (31). Exposure to CO levels at urbanized regions has also been demonstrated to have an association with reduced myocardial perfusion reserve, implying coronary endothelial dysfunction (32). Furthermore, CO leads to atherogenic processes both in coronary arteries and in peripheral artery systems. Case reports on acute CO poisoning have been shown to result in subsequent arterial thrombosis in the intracardiac region, and there have been studies reporting hypercoagulative states in patients with CO exposure, leading to development of atherosclerotic or thrombotic diseases (33).

PM_2.5_ exposure was also indicated as a risk factor for PAOD through our analysis. Oxidative stress, systemic inflammation, endothelial dysfunction, atherothrombosis, and arrhythmogenesis (18) are pathways that have been linked between PM_2.5_ and CV mortality. PM_2.5_ can lodge in the blood stream through the smallest airways and alveoli, penetrating the alveolar-capillary membrane, thereby leading to a high risk of weakening and rupture of vessels and to potential CV events.

Levels of CO and NO_2_ are mainly attributed to emissions from vehicles, especially in urbanized places. Thermal power plants located in central Taiwan are responsible for particulate matter in the air; however, they are carried north, to Taipei, by the wind (34). Several other factors, such as the southwesterly monsoonal flow and northeasterly monsoonal flow, and geographical division, have an influence on the PM_2.5_ concentrations. Taiwan is an island with a central mountain range running from north to south, and the complex topographical and geographical diversification leads to variations in PM_2.5_. Furthermore, PM_2.5_ is affected by the seasons, and its concentration starts to rise in autumn (September, October, and November) and winter (November, December, and January) until it peaks in spring (February, March, and April) and subsequently declines to a minimum during summer (May, June, and July). Hence, individuals' exposure to pollution was quantified using all the above factors through their residential areas along with the corresponding concentrations of NO_2_, PM_2.5_, and CO (Q1–Q4). This diversity in pollution among residential areas explains the differences in incident PAOD among geographical locations.

Therefore, based on the findings from this study, measures are required to minimize air pollution exposure and effective national pollution control plans need to be implemented in Taiwan. Physical activities of the elderly, children, and the general public should be strictly practiced in parks and gardens instead of primary traffic areas. Limiting outdoor activities during peak traffic hours, and wearing masks while outdoors, should be practiced at the individual level. Lastly, proper advice by health professionals regarding healthy living should be implemented. To have a comprehensive understanding of the far-reaching adverse effects of air pollution, follow-up studies will be conducted to fathom the effects of exposure to air pollutants on CVDs and other major health-related outcomes, for the Taiwanese population.

This study has several limitations. PAOD is based on the ICD-9-CM codes, which could be incorrect, hence there could be some misclassifications. However, prior studies on national cohorts have validated ICD-9-CM codes for diagnosis of chronic diseases (35–39). Moreover, the coverage of NHIRD is representative of the general population of Taiwan because of the universal reimbursement policy as operated by the government. The NHI Bureau of Taiwan also conducts extensive reviews of medical charts and imposes heavy penalties for any form of malpractice. In light of such strict measures, all ICD-9-CM codes for determination of PAOD are recorded as accurately as possible by the physicians after extensive assessments during the reimbursement process based on clinical and laboratory data. A second limitation of this study is the unavailability of some crucial CV-related parameters in the NHIRD, such as smoking status, body mass index (BMI), obesity, alcoholism, exercise, and dietary habits, which have been established as strong risk factors for PAOD (40). This was important, as ~25% of the population in Taiwan is obese, and a similar proportion of people use tobacco products. Also, prior studies have established smoking to be a strong risk factor for PAOD (41). To try to account for the effect of BMI and obesity, we have adjusted our model using hypertension, DM, and hyperlipidemia in our analysis. Furthermore, to adjust for the effects of smoking, we have incorporated smoking-related disorders such as COPD and cerebral vascular disease (stroke) in our fitted model.

## Conclusion

This study showed that, in addition to well-known risk factors, PAOD exhibits a high risk of incidence in a 10-year follow-up period for subjects undergoing prolonged exposure to air pollutants such as PM_2.5_ and CO. Findings from this study could be used to inform governmental institutions to develop effective pollution control plans to not only reduce the risk of overall incidence of PAOD but to also reduce the eventual risk of CV morbidity and mortality in an aging society such as Taiwan.

## Data Availability Statement

Data are available from the National Health Insurance Research Database (NHIRD) published by Taiwan National Health Insurance Administration (NHIA). Due to legal restrictions imposed by the government of Taiwan in relation to the “Personal Information Protection Act”, data cannot be made publicly available. Requests for data can be sent as a formal proposal to the NHIRD (http://nhird.nhri.org.tw). The authors did not have any special access privileges that others would not have.

## Ethics Statement

The studies involving human participants were reviewed and approved by Taichung Veterans General Hospital, Taichung, Taiwan. The patients/participants provided their written informed consent to participate in this study. Written informed consent was obtained from the individual(s) for the publication of any potentially identifiable images or data included in this article.

## Author Contributions

M-SH, S-HL, and C-SC: conceptualization and writing—original draft. S-HL and C-SC: formal analysis, investigation, and methodology. M-SH, L-HJ, S-YH, C-KH, and VH: project administration. C-SC: resources. M-SH, L-HJ, and C-SC: supervision. C-KH: validation. M-SH, L-HJ, C-KH, and VH: writing—review and editing. All authors have read and approved the manuscript for publication.

## Funding

This work was supported by grants from the Taichung Veterans General Hospital (TCVGH), Taiwan (TCVGH-1077329D and TCVGH-1077311C), and the Taipei Veterans General Hospital, Taoyuan branch, Taiwan (TYVH-10808, TYVH-10809, and TYVH-10902). The funders had no role in the study design, data collection, analysis, decision to publish, or preparation of the manuscript.

## Conflict of Interest

The authors declare that the research was conducted in the absence of any commercial or financial relationships that could be construed as a potential conflict of interest.

## Publisher's Note

All claims expressed in this article are solely those of the authors and do not necessarily represent those of their affiliated organizations, or those of the publisher, the editors and the reviewers. Any product that may be evaluated in this article, or claim that may be made by its manufacturer, is not guaranteed or endorsed by the publisher.
